# Breathalysing and surveying river users in Australia to understand alcohol consumption and attitudes toward drowning risk

**DOI:** 10.1186/s12889-018-6256-1

**Published:** 2018-12-19

**Authors:** Amy E. Peden, Richard C. Franklin, Peter A. Leggat

**Affiliations:** 1 Royal Life Saving Society – Australia, PO Box 558, Broadway, NSW 2007 Australia; 2 College of Public Health, Medical and Veterinary Sciences, Division of Tropical Health and Medicine, James Cook University, Townsville, Queensland 4811 Australia; 3 School of Public Health, Faculty of Health Sciences, University of the Witwatersand, Johannesburg, South Africa

**Keywords:** Drowning, Alcohol, Breathalysing, Exposure, Injury, Public health, Health promotion

## Abstract

**Background:**

Little is known about people’s river usage, a leading drowning location. This study examines alcohol consumption patterns of river users and their attitudes to drowning risk.

**Methods:**

A convenience sample of adult (18+ years) river users were surveyed at four river locations. The survey covered eight domains: demographics; river attendance frequency; frequency of engaging in water activities; drinking patterns; alcohol and water safety knowledge; alcohol and water safety attitudes; alcohol consumption; and Blood Alcohol Concentration (BAC). For BAC, participants were asked to record time since their last alcoholic drink and were then breathalysed to record an estimate of their BAC. BAC was examined by BAC reading (negative, positive, ≥0.050%). Hazardous lifetime drinking levels were calculated and their impact on drowning risk evaluated. Univariate and chi square analysis (95% confidence interval) was conducted.

**Results:**

Six hundred eighty four people participated (51.6% female; 49.0% aged 18–34 years). Sixteen percent (15.9%) had a positive BAC (Mean + BAC = 0.068%; SD ± 0.08; Range = 0.001–0.334%), with 7.2% ≥0.050% (Mean BAC ≥0.050% =0.132%; SD ± 0.06). Those significantly more likely to record a BAC ≥0.050% at the river were: aged 18–34 years, resided in inner regional and low socio-economic areas, visited the river in the afternoon, with friends, on days with higher maximum air temperatures, frequent river users (11+ times in the last 30 days) and those who spend longer in the water (301+ minutes). River users who recorded a BAC ≥0.050% were more likely to self-report engaging in risky activities (i.e. diving into water of unknown depth and jumping into the river from height). River users on Australia day (a national public holiday) were significantly more likely to drink heavily (Mean BAC ≥0.05% = 0.175%; SD ± 0.09).

**Conclusions:**

Despite males accounting for 85% of alcohol-related river drowning deaths, similar numbers of males and females were consuming alcohol at the river. This study has addressed a gap in knowledge by identifying river usage and alcohol consumption patterns among those at increased drowning risk. Implications for prevention include delivering alcohol-related river drowning prevention strategies to both males and females; at peak times including during hot weather, afternoons, public holidays and to river users who swim.

**Electronic supplementary material:**

The online version of this article (10.1186/s12889-018-6256-1) contains supplementary material, which is available to authorized users.

## Background

Alcohol is a known risk factor for drowning (both fatal and non-fatal) and aquatic-related injury [1–4] with up to 41% of river drowning deaths involving alcohol [5]. Alcohol disproportionately affects drowning risk in males [6–8], boating-related incidents [1, 9–11], natural waters [8, 10] and among Indigenous populations [5]. Globally, drowning is estimated to claim the lives of 372,000 people per annum [12], a statistic that is likely to underreport the true burden [13]. In Australia, an average of 281 people per year die from unintentional drowning. While the fatal drowning rate has reduced by 28% in Australia since 2002/03 [14], largely driven by reductions among children under five [15], the number of people drowning in rivers has stayed persistently high [11, 16].

In Australia, between 2002/03 and 2011/12, an average of 289 people died in Australia due to unintentional drowning [11]. Common fatal drowning scenarios in Australia include young children drowning unsupervised in bathtubs [17] and swimming pools [15] and adult males drowning in natural waterways such as beaches, oceans and in rivers [16] due to alcohol, pre-existing medical conditions [18] and not wearing a lifejacket [19]. Rivers are the leading location for drowning in Australia [11] with leading activities being undertaken prior to drowning including accidental falls into water (21.3%), non-aquatic transport incidents (commonly driving a motor vehicle into floodwaters) (18.2%) and swimming (16.2%). Alcohol is a known risk factor for unintentional fatal drowning in rivers in Australia, with the average adult drowning victim who had consumed alcohol prior to death recording a blood alcohol concentration (BAC) of 0.200% [5], a figure which is four times the upper legal limit for operating a motor vehicle and powered vessel in Australia [20].

Australia is a country with widespread alcohol consumption [21]. In 2017, 37% of Australians reported drinking alcohol on a weekly basis, almost one in five (17%) exceed the lifetime alcohol risk guidelines (more than two standard drinks per day) and 16% drink at hazardous levels (i.e. usually consuming four or more standard drinks per day) [22]. Alcohol is second only to tobacco as a cause of drug-related death and hospitalisation, responsible for 5.1% of the total burden of disease and injury in Australia in 2011 [21]. Each week, on average, more than 100 Australians die and more than 3000 are hospitalised as a result of excessive alcohol consumption [23]. People who drink regularly at higher levels place themselves at increased risk of chronic ill health and premature death [21, 22, 24], including due to injuries such as drowning [5].

Alcohol increases drowning risk due to its effects on cognitive processing, central nervous system processing, and physiological responses [2]. Alcohol is a vasodilator increasing the period of time someone may choose to remain in cold water thus increasing the risk of hypothermia [25]. Alcohol also causes labyrinthine dysfunction leading to decreased balance and impaired hearing and it also impairs judgement increasing the likelihood of exposure to high-risk situations [26]; all of which contribute to increased drowning risk [2–5]. In Australia, alcohol-related unintentional river drowning fatalities are significantly more likely as a result of jumping in, among those who identify as Aboriginal and Torres Strait Islander and those who drown in the evening (6:01 pm to 12 am) and early morning (12:01 am to 6 am) hours [5].

Previously published research on river drowning has identified the need for exposure studies [5, 11, 27] and real-time data collection in the field [28]. Given the role of alcohol in fatal river drowning, there is a need to understand alcohol consumption patterns of river users, as well as attitudes to alcohol consumption and aquatic activity. Breathalysers, which estimate a person’s BAC indirectly by measuring the alcohol on the breath, have predominately been used in broader injury research [29, 30], including road traffic-related injury [31, 32]; however only one drowning-related study has been conducted using breathalysers at Australian beaches [33]. While self-reported surveys provide an indication of the amount of alcohol consumed, breathalysing is a robust, objective measure of alcohol concentration [34], which has shown to give a reliable estimation of BAC [35].

Little is known about those who visit rivers, including demographics, activities being undertaken and exposure to drowning risk [5, 11, 27]. One previously published study, which used a survey of self-reported river exposure to re-calculate fatal river drowning rates based on exposure in Australia, found that males and females visited the river in similar proportions in a year (males 74.7% and females 72.2%) albeit for different activities; females significantly more likely to visit the river for non-aquatic activities (55.6%; *p* < 0.001) such as picnics and walking beside the river and males for fishing (11.9%; *p* = 0.001) and watercraft-related activities (7.1%;*p* = 0.020) [28]. Sixteen percent of those surveyed also reported consuming alcohol at the river [28].

Given the influence of alcohol in fatal river drowning, this study specifically focuses on the self-reported drinking patterns of river users as well as alcohol consumption on the day surveyed. This study aimed to describe the demographic profile of river users, explore attitudes toward river safety and alcohol use at rivers and measure the BACs of river users at a point in time. The study will also discuss considerations for the prevention of alcohol-related river drowning.

## Methods

### Study design

A cross-sectional convenience sample of adult (18 years and older) river users were surveyed. People aged 18 years and over were chosen in accordance with ethical approval for the study design in that it only breathalysed adults. The survey featured a total of 34 questions across eight domains: demographics, frequency of river attendance, frequency of engaging in water activities, drinking patterns, knowledge (alcohol and water safety), attitudes (alcohol and water safety), alcohol consumption and BAC. Although the bulk of the survey was developed for this research, the survey domains of demographics, frequency of attendance at an aquatic location, and alcohol consumption and BAC have been previously been used in a study conducted at beaches [33]. The survey questionnaire was piloted prior to use in the field by researchers AEP and RCF. It was then also piloted by three colleagues not part of the research team. Minor modifications between the pilot and final survey phases were made, mainly moving questions to enhance the flow of the survey and modifying response categories to ensure adequate options were provided. The full survey can be viewed in Additional file 1: Understanding water safety at rivers survey form.

Potential respondents were approached, or approached the researchers, and asked to participate. The project was verbally explained to potential respondents and they were then provided with an information sheet that described the study and the ethics approval granted. If they were willing to participate, respondents noted their written informed consent (yes/no) in the first question of the survey. Respondents who completed the survey were invited to enter the draw for a $100 pre-paid credit card. Prize winner details were captured separately to survey responses. Four researchers collected data across the sites with predominately two collecting data at one time. The research team collaborated to ensure that a person was breathalysed or surveyed only once. Details on how the survey was administered (i.e. both electronically and on paper) have been published previously [36, 37].

Each paper-based survey was linked to the ID number for the entry generated by SurveyGizmo ™ to allow for cross-referencing if required. The survey instrument collected time of day the survey was commenced and also the time of day the BAC reading was recorded. All data collected were de-identified. If any potential respondents were deemed too intoxicated to give informed consent and/or complete the survey, the interview was ended and the potential participant was not invited to participate. There were no people surveyed and breathalysed who were deemed too intoxicated to give informed consent. They were also advised not to enter the water due to being at increased risk of drowning and injury.

### Study setting

**Fig. 1:**
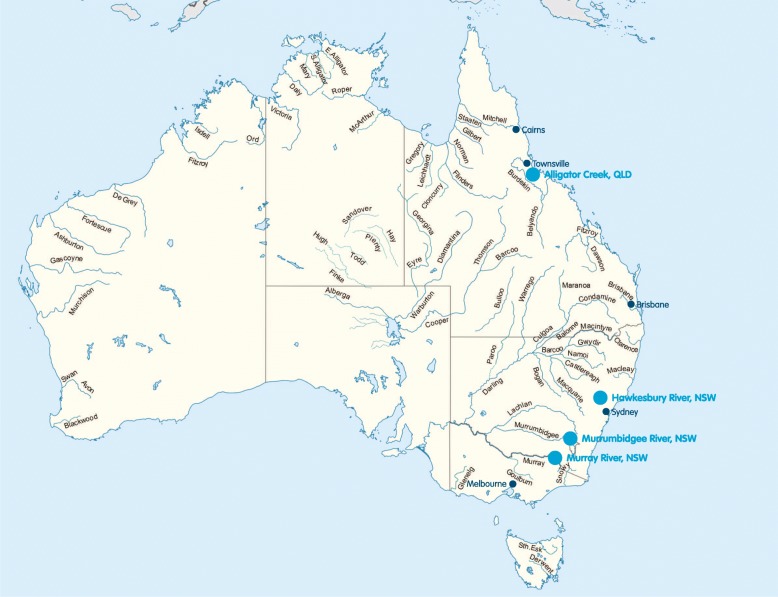
Map of Australia depicting the four pilot research sites. Please note: This figure has been adapted from the original map ‘Major rivers of Australia’ which is made freely available for use and adaptation under Creative Common license (CC BY-SA 3.0). The original map was created by Wikimedia Commons User Summerdrought. The original map can be found at the following address: (https://en.wikipedia.org/wiki/List_of_rivers_of_Australia#/media/File:Australian_rivers_with_names.png). The creator “Summerdrought” retains copyright and was not involved in the adaptations made to the map for this study.

Surveys were conducted at four high-risk river drowning locations namely Alligator Creek, the Murrumbidgee, Murray and Hawkesbury Rivers (Fig. 1). A detailed description of the research sites including site characteristics, data collection dates and weather data [38] (maximum air temperature and total daily rainfall) can be found in Additional file 2. This study forms part of a broader suite of work examining the epidemiology, risk factors and strategies for the prevention of river drowning in Australia [5, 11, 27, 28, 36, 37, 39, 40].

Data were collected across a 3 day period for three sites (Friday, Saturday and Sunday) and across a 7 day period (Monday to Sunday) at the Murray River. The Murray River data collection timeframe included Australia Day (Friday 26th January) which is a national public holiday in Australia. Due to the public holiday, the Murray River site was a designated ‘alcohol free zone’ by the local council and nominally enforced by police. At all other times and at all other sites, there was nothing in place regulating alcohol consumption beyond the requirement at Alligator Creek that no glass be taken onto the site.

Data collection occurred during the Australian summer (December–February inclusive) and during daylight savings (where the sun does not set until 8-9 pm at night). Data collection at all sites except the Hawkesbury River occurred during school holidays. To examine how river visitation, usage and alcohol consumption varied due to air temperature and rainfall, weather data were captured retrospectively from the Australian Bureau of Meteorology for each site for the days of data collection (Additional file 2: Table detailing characteristics of research sites, date of data collection, maximum air temperature and total daily rainfall).

### BAC testing

BAC readings were captured using LION Alcometers (LION SD400 ™), Lion Laboratories, United Kingdom. Two devices were available to maximise data collection. Devices were calibrated by Pacific Data Solutions (Ltd) prior to data collection commencing.

The respondent was required to exhale into a straw attached to the device for a continuous period of time (generally 5–10 s) until a BAC was recorded. A clean straw was used for each participant. The BAC reading was recorded as a continuous variable to three decimal places (e.g. 0.123%). The researcher administered the breathalyser and recorded BAC reading and time of day of the reading. Those who were drinking alcohol when approached by the research team were instructed not to drink while completing the survey; thereby allowing for approximately a 10 min period prior to being breathalysed where they did not consume alcohol. Those who recorded a BAC of ≥0.050% were advised by the researcher against going back in the water due to their increased risk due to intoxication.

### Data cleaning, coding, checking and statistical analysis

The final dataset of survey responses was downloaded from SurveyGizmo into IBMSPSS V20 [41] for data cleaning, coding, checking and analysis. The process of checking data transferred from paper-based surveys to the electronic database has been published previously [36, 37]. The four responses where a BAC reading was not captured were excluded from the dataset. Two responses where age of respondent was not captured were also excluded.

Age in years of respondent was coded into the following age bands to allow for comparison with previously collected data [5, 28]: 18–34 years; 35–54 years; and 55+ years. Time of day of BAC reading was recorded and BAC readings were cleaned to ensure consistency of format (e.g. time of day recoded into HH:MM using 24 h time) prior to analysis. Information on how the remoteness classification [42] and relative socio-economic status [43] of the survey respondents’ postcode was coded have been previously published [36, 37].

The activity of ‘recreate’ beside water relates to activities undertaken for leisure purposes, such as picnics, reading a book, sun bathing etc. For the question regarding frequency of engaging in water activities at rivers in the last 12 months, answers were converted into a dichotomous variable with ‘sometimes’ and ‘always’ recoded as ‘yes’ and ‘never’ and ‘N/A – Don’t do this’ recoded as no. Those with a blank response were removed prior to analysis.

Attitudinal questions required respondents to indicate their level agreement with statements on a five point Likert scale (strongly agree, agree, neither agree nor disagree, disagree, and strongly disagree) with a ‘don’t know’ option. For ease of analysis, ‘strongly agree’ and ‘agree’ were combined into ‘agree’ and ‘disagree’ and ‘strongly disagree’ were combined into ‘disagree’). This left four categories for analysis: agree, neither agree nor disagree, disagree and don’t know. Attitudinal questions asked included: “It’s okay to drink alcohol on a boat (as the skipper)” and “It’s okay to drink alcohol before swimming”. Knowledge based questions included: “When was the last time you undertook/updated first aid qualifications (including CPR)?”

Maximum daily air temperature were reported in quartiles as determined by IBM SPSS ™. For each upper end of the quartile (e.g. 24.7–31.5) this was rounded up to be displayed as (24.7–31.9). Temperature was recorded in degrees Celsius and was subsequently converted into degrees Fahrenheit. Both are displayed for ease of understanding for an international readership. Temperature quartiles used are: < 32.0 °C (< 89.6 °F); 32.0 °C–35.9 °C (89.6 °F–96.6 °F); 36.0 °C–39.9 °C (96.8 °F–103.8 °F); ≥40.0 °C (≥104.0 °F). Time of day of BAC reading was coded into morning (6:01 am to 12 pm), afternoon (12:01 pm to 6 pm) and evening (6:01 pm to 12 am).

This study examined the role of alcohol in two ways. Alcohol consumption on the day was measured using BAC and hazardous lifetime alcohol use was calculated using the Alcohol Use Disorder Identification Test (AUDIT) [44, 45]. Using self-reported data on alcohol consumption, an audit score was calculated as follows:

- Number of days in which you had at least one drink of any alcoholic beverage during the past 30 days. It was codified as 0 = none; 1 = 1 day; 2 = 2–4 days; 3 = 5–15 days; 4 = 16–20 days; and 5 = 24+ days.
- On the days when you drank, number of drinks on average during the past 30 days. Codified as 0 = between 0 and 2 drinks; 1 = between 3 and 4 drinks; 2 = between 5 and 6 drinks; 3 = between 7 and 9 drinks; and 4 = if ≥10 drinks.
- Considering all types of alcohol beverages, number of times in the past 30 days you have had 4 or more drinks (for females) or 6 or more drinks (for males) on a single occasion. Codified as 0 = none/less than monthly; 2 = between 1 and 7 times; 3 = between 8 and 12 times; 4 = if ≥13 times.

Hazardous alcohol use was calculated by adding the AUDIT scores as codified above (a + b + c). Alcohol use was considered hazardous if the resulting score was ≥3 in females and ≥ 4 in males [44, 45].

A contributory level of alcohol was defined as a BAC of ≥0.050% due to known impacts on decision making, motor skills and being the legislated upper limit for operating a motor vehicle and watercraft in most states and territories in Australia [2, 20]. For the purposes of analysis BAC readings were divided into three categories: BAC – No (a BAC of 0.000%), BAC between 0.001and 0.049%, and a BAC of ≥0.050%.

Univariate and chi-square analysis was undertaken with a 95% confidence interval. Chi square and non-parametric testing was undertaken to compare the distribution of survey responses collected by sex and demographic variables. Non-parametric testing was undertaken using the proportional basis of the Australian population as the assumed outcome numbers. Population data were sourced from the Australian Bureau of Statistics (ABS) using the most recent data available (September 2017) [46]. For cells with small counts (i.e. < 5) a Fisher’s Exact Test was used.

### Ethics

Ethics approval for this study was granted by the James Cook University Human Research Ethics Committee (HREC – H7249).

## Results

A total of 690 people were surveyed. After removing entries without BACs (*n* = 4) and without age recorded (*n* = 2), there remained a total of 684 responses included for analysis. Females accounted for 51.6% of the sample and 49.0% of the sample were people aged 18–34 years. Twelve (*n* = 1.8%) survey respondents were international tourists. The largest number of respondents was recorded at the Murray River (*n* = 278; 40.6%), followed by the Murrumbidgee River (*n* = 174; 25.4%), Alligator Creek (*n* = 120; 17.5%) and the Hawkesbury River (*n* = 112; 16.4%). Those surveyed at the Hawkesbury River were significantly more likely to be male (X^2^ = 46.0; *p* < 0.001), whereas the cohort surveyed at the Murrumbidgee (X^2^ = 3.9; *p* = 0.049) and Murray rivers (X^2^ = 5.9; *p* = 0.016) were significantly more likely to be female. Age group and Index of Relative Socio-economic Advantage and Disadvantage (IRSAD) of respondent’s residential postcode did not vary by sex of respondent (Table 1).

**Table 1:** Demographics of river users surveyed (*N* = 684)

	Total		Male		Female		X^2^ (*p* value)
	N	%	N	%	N	%	
Total	684	100.0	331	48.4	353	51.6	0.399 (*p* = 0.527)
Age group							
18–34 years	335	49.0	162	48.4	173	51.6	0.000 (*p* = 0.986)
35–54 years	251	36.7	120	47.8	131	52.2	0.054 (*p* = 0.816)
55+ years	98	14.3	49	50.0	49	50.0	0.118 (*p* = 0.731)
Remoteness classification of respondent’s residential postcode							
Major Cities	123	18.0	81	65.9	42	34.1	17.308 (*p* < 0.001)
Inner Regional	391	57.2	169	43.2	222	56.8	12.547 (*p* < 0.001)
Outer Regional, Remote & Very Remote	146	21.3	73	50.0	73	50.0	2.343 (*p* = 0.343)
Unknown/International	24	3.5	8	33.3	16	66.7	–
Country of Birth							
Australia	577	84.4	289	50.1	288	49.9	4.242 (*p* = 0.039)
Outside Australia	107	15.6	42	39.3	65	60.7	
IRSAD classification of respondent’s residential postcode							
Low	118	17.3	58	49.2	60	50.8	2.390 (*p* = 0.122)
High	113	16.5	67	59.3	46	40.7	
Other/Unknown/International	453	66.2	206	45.5	247	54.5	–

Please note: Chi square analysis excludes the other/unknown variables

The leading activities being undertaken at three of the sites (Alligator Creek, Murrumbidgee River and Murray River) were walk/sit/recreate beside the river (90.0% (*n* = 108) of respondents at Alligator Creek; 89.1% (*n* = 155) at Murrumbidgee River; and 93.2% (*n* = 259) at the Murray River), followed by swimming (85.8% (*n* = 103) of Alligator Creek respondents; 87.9% (*n* = 153) of Murrumbidgee River respondents; and 75.9% (*n* = 211) of Murray River respondents). The Hawkesbury river site differed in that the top two activities were boating (82.1% (*n* = 92) of respondents) and water skiing (54.5% (*n* = 61)).

Sixteen percent (15.9%; *n* = 109) respondents recorded a positive BAC reading when breathalysed (mean positive BAC = 0.068%; SD ± 0.08). A slightly higher proportion of females (16.1%) than males (15.8%) recorded positive BAC readings, however sex was not found to be statistically significant for consuming alcohol. People aged 18–34 years(X^2^ = 10.7; *p* = 0.001) and those residing in areas classified as Inner Regional (X^2^ = 9.0; *p* = 0.003) were significantly more likely to record a positive BAC reading; while those aged 55 years and older (X^2^ = 5.2; *p* = 0.023) and those residing in major cities (X^2^ = 7.3; *p* = 0.007) were significantly less likely to record a positive BAC. Australian-born respondents were more likely to record positive BAC readings (17.2% positive) compared to overseas born respondents (9.3% positive) (X^2^ = 4.1; *p* = 0.043). Respondents from postcodes classified as being low IRSAD were significantly more likely to record positive BAC readings (X^2^ = 5.7; *p* = 0.017), when compared to those residing in postcodes classified as high (Table 2).

**Table 2:** Blood alcohol concentration (BAC) readings by demographics of survey respondents and time of day (N = 684)

	Total		Alcohol Yes				Alcohol No		X^2^ (*p* value) comparing alcohol yes to alcohol no	X^2^ comparing BAC < 0.05% to BAC ≥0.05%
			BAC 0.001–0.049%		BAC ≥0.050%					
	N	%	N	%	N	%	N	%		
Total	684	100.0	60	8.8	49	7.2	575	84.1	–	–
Sex										
Male	331	48.4	25	7.6	27	8.2	279	84.3	0.024 (*p* = 0.876)	0.952 (*p* = 0.329)
Female	353	51.6	35	9.9	22	6.2	296	83.9		
Age group										
18–34 years	335	49.0	36	10.7	33	9.9	266	79.4	10.649 (*p* = 0.001)	7.128 (p = 0.008)
35–54 years	251	36.7	17	6.8	15	6.0	219	87.3	3.006 (*p* = 0.083)	0.841 (*p* = 0.359)
55+ years	98	14.3	7	7.1	1	1.0	90	91.8	5.158 (*p* = 0.023)	6.491 (*p* = 0.006)*
Remoteness classification of respondent’s residential postcode										
Major Cities	123	18.0	10	8.1	0	0.0	113	91.9	7.269 (*p* = 0.007)	12.124 (*p* < 0.001)*
Inner Regional	391	57.2	37	9.5	40	10.2	314	80.3	8.558 (*p* = 0.003)	10.989 (*p* = 0.001)
Outer Regional, Remote and Very Remote	146	21.3	11	7.5	9	6.2	126	86.3	0.872 (*p* = 0.350)	0.433 (*p* = 00.511)
Unknown/International	24	3.5	2	8.3	0	0.0	22	91.7	–	–
Country of Birth										
Australia	577	84.4	53	9.2	46	8.0	478	82.8	4.112 (*p* = 0.043)	3.626 (*p* = 0.065)*
Outside Australia	107	15.6	7	6.5	3	2.8	97	90.7		
IRSAD classification of respondent’s residential postcode										
Low	118	17.3	13	11.0	16	13.6	89	75.4	5.659 (*p* = 0.017)	16.462 (*p* < 0.001)*
High	113	16.5	14	12.4	0	0.0	99	87.6		
Other/Unknown/International	453	66.2	33	7.3	33	7.3	387	85.4	–	–
Time of day										
Morning (6:01 am to 12 pm)	196	28.7	11	5.6	0	0.0	185	94.4	21.855 (*p* < 0.001)	21.199 (*p* < 0.001)*
Afternoon (12:01 pm to 6 pm)	476	69.6	49	10.3	42	8.8	385	80.9	11.831 (*p* = 0.001)	6.484 (*p* = 0.011)
Evening (6:01 pm to 12 am)	12	1.8	0	0.0	7	58.3	5	41.7	16.390 (*p* < 0.001)	48.088 (*p* < 0.001)*

Please note: Chi square analysis excludes the other/unknown variables. *p value reported is Fisher’s Exact Test

Seven percent (7.2%) of respondents recorded a BAC ≥0.05%. The mean BAC ≥0.05% was 0.132% (SD ± 0.06; Range 0.001–0.334%). The mean BAC ≥0.05% for males was 0.129% (SD ± 0.09) and 0.136% (SD ± 0.08) for females. Respondents aged 18–34 years were significantly more likely to record a BAC ≥0.05% (X^2^ = 7.1; *p* = 0.008), while those aged 55 years and older were significantly less likely to (X^2^ = 6.5; *p* = 0.006).

**Fig. 2:**
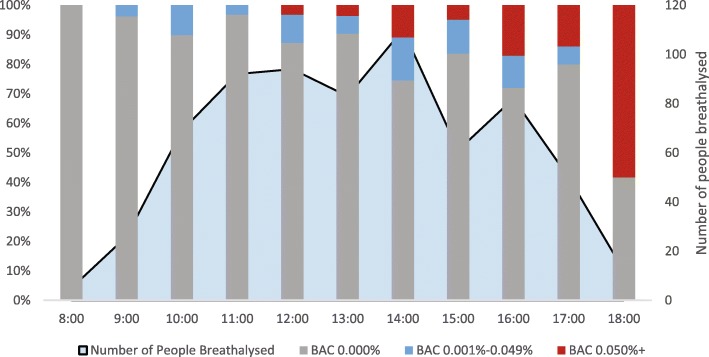
Blood alcohol concentration (BAC) ≥0.05% by time of day (*n* = 49)

Respondents residing in areas classified as major cities were significantly less likely to record a BAC ≥0.05% (X^2^ = 12.1; *p* < 0.001) while respondents from inner regional areas were significantly more likely to record a BAC ≥0.05% (X^2^ = 11.0; *p* = 0.001). Respondents from low IRSAD areas were more likely to record a BAC ≥0.05% (X^2^ = 16.5; *p* < 0.001) (Table 3). Respondents at the Hawkesbury River site, were significantly less likely to record a positive BAC (X^2^ = 15.3; p < 0.001) or a BAC ≥0.05% (X^2^ = 7.9; *p* = 0.005). All BAC readings ≥0.05%, were recorded in the afternoon (X^2^ = 6.5; *p* = 0.011) or evening (X^2^ = 48.1; p < 0.001) with over half (57.1%) being recorded between 4 pm and 6 pm (Fig. 2).

**Table 3:** Blood alcohol concentration (BAC) readings of river users by daily maximum temperatures, river attendance information and self-reported swimming ability, chi square (*N* = 684)

	Total		Alcohol Yes				Alcohol No		X^2^ (p value) comparing alcohol yes to alcohol no	X^2^ comparing over BAC < 0.05% to BAC ≥0.05%
			BAC 0.001–0.049%		BAC ≥0.050%					
	N	%	N	%	N	%	N	%		
Daily maximum air temperature degrees Celsius (Fahrenheit)										
< 32.0 (< 89.6)	182	26.6	6	3.3	1	0.5	173	95.1	22.361 (*p* < 0.001)	11.343 (*p* < 0.001)*
32.0–35.9 (89.6–96.6)	167	24.4	16	9.6	10	6.0	141	84.4	0.022 (*p* = 0.882)	0.459 (*p* = 0.498)
36.0–39.9 (96.8–103.8)	189	27.6	22	11.6	21	11.1	146	77.2	9.056 (*p* = 0.003)	6.119 (*p* = 0.013)
≥ 40.0 (≥104.0)	146	21.3	16	11.0	15	10.3	115	78.8	3.888 (*p* = 0.049)	2.700 (*p* = 0.100)
At river alone or with others										
Alone	41	6.0	2	4.9	0	0.0	39	95.1	3.981 (*p* = 0.046)	3.366 (*p* = 0.107)*
With friends	412	60.2	39	9.5	44	10.7	329	79.9	13.707 (*p* < 0.001)	19.257 (*p* < 0.001)
With family	317	46.3	22	6.9	11	3.5	284	89.6	13.466 (*p* < 0.001)	12.120 (*p* < 0.001)
Frequency of visiting any river in the last 30 days (month)										
1–2 times	248	36.3	19	7.7	21	8.5	208	83.9	0.004 (*p* = 0.947)	0.946 (*p* = 0.331)
3–5 times	166	24.3	15	9.0	6	3.6	145	87.3	1.838 (*p* = 0.175)	4.215 (*p* = 0.040)
6–10 times	128	18.7	10	7.8	8	6.3	110	85.9	0.443 (*p* = 0.506)	0.211 (*p* = 0.646)
11+ times	139	20.3	16	11.5	14	10.1	109	78.4	4.040 (*p* = 0.044)	2.164 (*p* = 0.141)
Unknown	3	0.4	0	0.0	0	0.0	3	100.0	–	–
Length of time spent in water (in minutes)										
0 min	152	22.2	15	9.9	3	2.0	134	88.2	2.445 (*p* = 0.118)	7.915 (*p* = 0.004)*
1–30 min	283	41.4	32	11.3	17	6.0	234	82.7	0.685 (*p* = 0.408)	0.971 (*p* = 0.324)
31–60 min	87	12.7	5	5.7	3	3.4	79	90.8	3.380 (*p* = 0.066)	2.069 (*p* = 0.184)*
61–120 min	53	7.7	4	7.5	7	13.2	42	79.2	0.996 (*p* = 0.318)	3.155 (*p* = 0.091)*
121–300 min	89	13.0	4	4.5	13	14.6	72	80.9	0.765 (*p* = 0.382)	8.523 (*p* = 0.007)*
301+ minutes	20	2.9	0	0.0	6	30.0	14	70.0	3.042 (*p* = 0.081)	16.155 (*p* = 0.002)*

Please note: Chi square analysis excludes the other/unknown variables. *p value reported is Fisher’s Exact Test

Respondents were significantly more likely to both record a positive BAC (X^2^ = 32.3; *p* < 0.001) and a BAC ≥0.05% (X^2^ = 32.0; *p* < 0.001) on the Australia Day public holiday. Twenty-six percent (26.4%) of river users breathalysed on Australia Day recorded a BAC ≥0.05% (Mean BAC ≥0.05% =0.175%; SD ± 0.09). The BACs recorded on Australia Day ranged from 0.000 to 0.308%.

Air temperature impacts alcohol consumption, with days recording cooler maximum air temperatures (< 32.0 °C) significantly less likely to have respondents recording positive BACs (X^2^ = 22.4; *p* < 0.001) or BACs ≥0.05% (X^2^ = 11.3; *p* < 0.001); whereas days with a higher maximum air temperature (36.8–39.9) being significantly more likely to have respondents with positive BACs (X^2^ = 9.1; *p* = 0.003) and BACs ≥0.05% (X^2^ = 6.1; *p* = 0.013) (Table 3).

Respondents visiting the river alone were significantly less likely to report a positive BAC (X^2^ = 4.0; *p* = 0.046). Respondents visiting the river with family were also significantly less likely to report a positive BAC (X^2^ = 13.5; *p* < 0.001) and a BAC ≥0.05% (X^2^ = 19.3; p < 0.001); whereas those visiting the river with friends were significantly more likely to both report a positive BAC (X^2^ = 13.7; *p* < 0.001) and a BAC ≥0.05% (X^2^ = 12.1; *p* < 0.001) (Table 3).

Thirty-six percent (36.3%) of respondents reported visiting a river 1–2 times in the last 30 days, with a further 24.3% visiting 3–5 times. Those visiting a river 3–5 times were significantly more likely to record a BAC ≥0.05% (X^2^ = 4.2; *p* = 0.040) whereas those visiting a river 11+ times were significantly more likely to record a positive BAC (X^2^ = 4.0; *p* = 0.044).

On average people spent 60.1 min in the water (SD ± 89.8) with 41 % (41.4%) of river users surveyed stated they spent 1–30 min in the water per visit. Respondents who did not enter the water were significantly less likely to record a BAC ≥0.05% (X^2^ = 7.9; *p* = 0.004), whereas those spending 301+ minutes in the water were significantly more likely to record a BAC ≥0.05% (X^2^ = 16.2; *p* = 0.002) (Table 3).

River users were asked to indicate how frequently they participated in a range of activities at any river in the last 12 months. When compared to BAC reading, those who stated they had participated in the alcohol-related aquatic activities (e.g. swimming within 2 h of consuming alcohol, boating within 2 h of consuming alcohol, either as the passenger or the skipper) were significantly more likely to record a positive BAC; swimming (X^2^ = 47.5; *p* < 0.001) and boating (X^2^ = 6.2; *p* = 0.013). Those who self-reported swimming alone were also significantly more likely to record a positive BAC (X^2^ = 6.1; *p* = 0.013) (Table 4).

**Table 4:** Self-reported frequency of undertaking risky/alcohol-related behaviours at rivers in the past 12 months and drinking alcohol prior to visiting and while at the river, by BAC and hazardous drinking levels, chi square

	Total		BAC 0.000		Positive BAC (0.001 and higher)		X^2^ comparing BAC 0.000 to positive BAC (0.001 and higher)	Hazardous drinking levels - Yes		Hazardous drinking levels - No		Hazardous levels - Unknown		X^2^ comparing hazardous drinking levels - yes to hazardous drinking levels - no
	N	%	N	%	N	%		N	%	N	%	N	%	
Swim within 2 h of consuming alcohol (*n* = 677)														
Yes	277	40.9	200	35.2	77	70.6	47.489 (*p* < 0.001)	227	52.4	45	19.2	5	50.0	69.309 (*p* < 0.001)
No	400	59.1	368	64.8	32	29.4		206	47.6	189	80.8	5	50.0	
Boat within 2 h of consuming alcohol (as passenger or skipper) (*n* = 676)														
Yes	166	24.6	129	22.8	37	33.9	6.183 (*p* = 0.013)	124	28.6	38	16.3	4	40.0	12.508 (*p* < 0.001)
No	510	75.4	438	77.2	72	66.1		309	71.4	195	83.7	6	60.0	
Swim alone (*n* = 674)														
Yes	351	52.1	283	50.0	68	63.0	6.107 (*p* = 0.013)	237	54.9	106	45.7	8	80.0	5.084 (*p* = 0.024)
No	323	47.9	283	50.0	40	37.0		195	45.1	126	54.3	2	20.0	
Jump into a river from a height (e.g. from tree, bridge, rocks or using a rope swing) (*n* = 677)														
Yes	232	34.3	193	34.0	39	35.8	0.132 (*p* = 0.717)	173	40.0	56	23.9	3	30.0	17.297 (*p* < 0.001)
No	445	65.7	375	66.0	70	64.2		260	60.0	178	76.1	7	70.0	
Swim in water that was too deep to touch the bottom (*n* = 677)														
Yes	512	75.6	424	74.8	88	80.7	1.838 (*p* = 0.175)	339	78.3	165	70.5	8	80.0	4.977 (*p* = 0.026)
No	165	24.4	144	25.4	21	19.3		94	21.7	69	29.5	2	20.0	
Dive into water of unknown depth (n = 676)														
Yes	155	22.9	124	21.9	31	28.4	2.234 (*p* = 0.135)	114	26.4	39	16.7	2	20.0	8.108 (*p* = 0.004)
No	521	77.1	443	78.1	78	71.6		318	73.6	195	83.3	8	80.0	
Camp (stay overnight) (*n* = 677)														
Yes	293	43.3	240	42.3	53	48.6	1.512 (*p* = 0.219)	203	46.9	84	35.9	6	60.0	7.477 (*p* = 0.006)
No	384	56.7	328	57.7	56	51.4		230	53.1	150	64.1	4	40.0	
How often is alcohol consumed prior to visiting a river (*n* = 680)														
Never	429	63.1	391	68.5	38	34.9	49.655 (*p* < 0.001)	227	52.3	199	84.3	3	30.0	75.033 (*p* < 0.001)
Sometimes	222	32.6	161	28.2	61	56.0	30.035 (*p* < 0.001)	187	43.1	30	12.7	5	50.0	63.114 (*p* < 0.001)
Always	15	2.2	5	0.9	10	9.2	28.366 (*p* < 0.001)	14	3.2	0	0.0	1	10.0	7.654 (*p* = 0.003)*
Not Applicable	14	2.1	14	2.5	0	0.0	–	6	1.4	7	3.0	1	10.0	–
How often is alcohol consumed while at the river (*n* = 680)														
Never	343	50.4	322	56.4	21	19.3	52.839 (*p* < 0.001)	169	38.9	171	72.5	3	30.0	74.559 (*p* < 0.001)
Sometimes	307	45.1	233	40.8	74	67.9	25.696 (*p* < 0.001)	244	56.2	58	24.6	5	50.0	59.136 (*p* < 0.001)
Always	21	3.1	7	1.2	14	12.8	40.509 (*p* < 0.001)	19	4.4	1	0.4	1	10.0	8.047 (*p* = 0.005)*
Not Applicable	9	1.3	9	1.6	0	0.0	–	2	0.5	6	2.5	1	10.0	–

*p value reported is Fisher’s Exact Test

Sixty-four percent (63.6%) of river users surveyed were found to consume alcohol at hazardous levels. River users who self-reported participating in all activities were significantly more likely to be drinking at hazardous levels (*p* < 0.05), with results most pronounced for the activities of swimming within 2 h of consuming alcohol (X^2^ = 69.3; *p* < 0.001), boating within 2 h of consuming alcohol (as passenger or skipper) (X^2^ = 12.5; *p* < 0.001) and jumping into a river from a height (X^2^ = 17.3; *p* < 0.001) (Table 4).

Thirty-five percent (34.8%) of respondents stated they sometimes (32.6%) or always (2.2%) consumed alcohol prior to visiting a river. Those who responded sometimes or always to this question were significantly more likely to record a positive BAC (sometimes X^2^ = 30.0; *p* < 0.001 and always X^2^ = 28.4; *p* < 0.001). Forty-eight percent (48.2%) of respondents stated they sometimes (45.1%) or always (3.1%) consumed alcohol while at the river. Those who responded both sometimes (X^2^ = 25.7; *p* < 0.001) and always (X^2^ = 40.5; *p* < 0.001) to this question were significantly more likely to record a positive BAC when breathalysed. Respondents with hazardous alcohol consumption levels were significantly more likely to self-report always (X^2^ = 7.7; *p* = 0.003) or sometimes (X^2^ = 63.1; *p* < 0.001) drinking alcohol prior to visiting a river and always (X^2^ = 8.1; *p* = 0.005) or sometimes (X^2^ = 59.1; *p* < 0.001) consuming alcohol when at the river (Table 4).

River users were also asked attitudinal questions related both to alcohol and driving a motor vehicle, as well as specific aquatic-related questions (alcohol and boating; alcohol and swimming). Those who recorded a positive BAC when breathalysed at the river were significantly more likely to agree that it is okay to drink alcohol on a boat as a passenger (X^2^ = 7.9; p = 0.005), that it’s okay to drink alcohol on a boat as the skipper (X^2^ = 10.0; *p* = 0.002) and to drink alcohol before swimming (X^2^ = 13.3; *p* < 0.001) (Table 5).

**Table 5:** Attitudinal questions related to alcohol by BAC and hazardous drinking levels, chi square (*N* = 684)

	Total		BAC 0.000		Positive BAC (0.001 and higher)		X^2^ comparing BAC 0.000 to positive BAC (0.001 and higher)	Hazardous drinking levels - Yes		Hazardous drinking levels - No		Hazardous levels - Unknown		X^2^ comparing hazardous drinking levels - yes to hazardous drinking levels - no
	N	%	N	%	N	%		N	%	N	%	N	%	
Total	684	100.0	575	84.1	109	15.9	–	435	63.6	236	34.5	13	1.9	–
It’s okay to drink alcohol and drive a motor vehicle														
Agree	49	7.2	38	6.6	11	10.1	1.663 (*p* = 0.197)	31	7.1	17	7.2	1	7.7	0.000 (*p* = 0.984)
Neither agree nor disagree	31	4.5	24	4.2	7	6.4	1.064 (*p* = 0.302)	23	5.3	7	3.0	1	7.7	1.965 (*p* = 0.161)
Disagree	597	87.3	507	88.2	90	82.6	2.900 (*p* = 0.089)	377	86.7	211	89.4	9	69.2	0.789 (*p* = 0.374)
Don’t Know	7	1.0	6	1.0	1	0.9	–	4	0.9	1	0.4	2	15.4	–
It’s okay to drink alcohol on a boat (as a passenger)														
Agree	287	42.0	228	39.7	59	54.1	7.873 (*p* = 0.005)	213	49.0	67	28.4	7	53.8	28.508 (*p* < 0.001)
Neither agree nor disagree	135	19.7	116	20.2	19	17.4	0.453 (*p* = 0.501)	86	19.8	48	20.3	1	7.7	0.007 (*p* = 0.932)
Disagree	248	36.3	219	38.1	29	26.6	5.366 (*p* = 0.021)	126	29.0	119	50.4	3	23.1	29.061 (*p* < 0.001)
Don’t Know	14	2.0	12	2.1	2	1.8	–	10	2.3	2	0.8	2	15.4	–
It’s okay to drink alcohol on a boat (as the skipper)														
Agree	68	9.9	48	8.3	20	18.3	10.028 (*p* = 0.002)	52	12.0	14	5.6	2	15.4	6.409 (*p* = 0.011)
Neither agree nor disagree	48	7.0	38	6.6	10	9.2	0.879 (*p* = 0.349)	36	8.3	11	4.7	1	7.7	3.158 (*p* = 0.076)
Disagree	557	81.4	479	83.3	78	71.6	10.021 (*p* = 0.002)	340	78.2	209	88.6	8	61.5	10.426 (*p* = 0.001)
Don’t Know	11	1.6	10	1.7	1	0.9	–	7	1.6	2	0.8	2	15.4	–
It’s okay to drink alcohol before swimming														
Agree	142	20.8	105	18.3	37	33.9	13.308 (*p* < 0.001)	106	24.4	34	14.4	2	15.4	9.355 (*p* = 0.002)
Neither agree nor disagree	133	19.4	104	18.1	29	26.6	4.041 (p = 0.044)	99	22.8	33	14.0	1	7.7	7.592 (*p* = 0.006)
Disagree	397	58.0	355	61.7	42	38.5	21.694 (*p* < 0.001)	223	51.3	166	70.3	8	61.5	22.827 (*p* < 0.001)
Don’t Know	12	1.8	11	1.9	1	0.9	–	7	1.6	3	1.3	2	15.4	–

Please note: Chi square analysis excluded the ‘don’t know’ variable

Those with hazardous drinking levels were significantly more likely to agree with the statements ‘it’s okay to drink alcohol on a boat as a passenger’ (X^2^ = 28.5; *p* < 0.001), ‘it is okay to drink alcohol on a boat as the skipper’ (X^2^ = 6.4; *p* = 0.011) and ‘it’s okay to drink alcohol before swimming’ (X^2^ = 9.4; *p* = 0.002) (Table 5).

## Discussion

Alcohol is a leading risk factor for fatal unintentional drowning in rivers in Australia [5]. Sixteen percent of river users recorded a positive BAC, with 7% of these recording a contributory level of alcohol (BAC ≥0.05%). Sixty-four percent (63.6%) of river users surveyed were found to consume alcohol at hazardous levels, compared to 18% of the Australian population aged 18 years and over in 2016 [22].

River users residing in inner regional areas, areas defined as low IRSAD, who visit the river in the afternoon, with friends, on days with higher maximum air temperatures, frequent river users (11+ times in the last 30 days) and those who spend longer on average in the water (301+ minutes) were significantly more likely to have contributory levels of alcohol when breathalysed. Key findings with a focus on comparisons with previously published alcohol-related fatal drowning statistics and river exposure are discussed, as well as implications for river drowning prevention.

### Alcohol consumption

A previously conducted nationally representative computer-assisted telephone interviewing (CATI) survey of river users found that 16% of people surveyed self-reported consuming alcohol at a river when they visit [28]. Similarly, this study found 16% of those surveyed recorded positive BACs at the river when breathalysed. When comparing the two studies by sex and age group, 9% of females and 15% of males self-reported consuming alcohol at the river, compared to 16% of males and females respectively when breathalysed, indicating females may underreport their alcohol consumption at rivers when asked to self-report [28].

There are inconsistencies in the drinking behaviour of river users when compared to the general population. This study found 13% of river users drink five or more alcoholic drinks per day, compared with 7% of the Australian population [47]. With respect to river users who drink at risky levels, 24% of river users surveyed stated they did, similar to 26% of the Australian population [21]. These findings suggest river users surveyed are twice as likely to drink at heavier levels daily than the general population, but are not binge drinking as much as the general population. Reducing alcohol-related drowning risk among this cohort of river users will be challenging, and starts with behaviour change in daily life, far removed from river drowning risk. This behavior is also carried into the river setting and work is required to ensure that the activity of drinking and entering the water is avoided.

### Sex differences

Males continue to be the primary target of strategies aimed at reducing drowning at river locations in Australia [48]. This is warranted as males account for the vast majority (84%) of river drowning deaths where blood alcohol levels are known to be contributory [5]. The authors note, however, that the females breathalysed in this study, are drinking at similar rates as males. Females accounted for 45% of all river users with a BAC ≥0.05%, recorded a higher mean BAC ≥0.05% (0.139%) than males (0.129%) and a higher number of females (*n* = 18) than males (*n* = 15) recorded a BAC of ≥0.100% (double the contributory level). These findings are supported by recent research that identifies rates of alcohol use appear to be converging among males and females [49], with more females in younger cohorts increasingly likely to record higher levels of alcohol use and abuse [50]. Despite decreases among Australian males, exceeding lifetime alcohol risk guidelines in females has remained similar [22].

Further research is warranted to examine the differences in behaviour (and the factors underpinning this) that see males and females drink at equally risky levels, but predominately males represented in fatal river drowning statistics where alcohol is involved. With clear links identified between masculinity and risky drinking behaviours around water [51, 52], the authors postulate that males may be pressured to go back into the water and engage in risky behaviours after consuming alcohol, whereas females may be more likely to stay on the bank when under the influence of alcohol. This assumption requires further testing to examine the different attitudes between males and females influencing this behaviour. Further research should also be conducted to test this study’s findings of alcohol consumption (and BAC levels) among females at more river locations.

### Time of day

People who were surveyed and breathalysed at rivers in the afternoon and evening hours were significantly more likely to record BACs ≥0.05%. This mirrors analysis of fatal river drowning data in Australia that shows 64.3% of all fatal river drowning with a contributory level of alcohol occurred at such times. Evening hours show a link between fatal river drowning and contributory levels of alcohol [5], posing a challenge for data collection. The number of people at rivers in the evening hours is scarce, however the likelihood of recording a positive BAC increased, mirroring the number of alcohol-related drowning deaths at these hours [5]. Alcohol related drowning deaths at rivers in the evening appear to be a rare yet regularly occurring event and as such, prevention of such drowning deaths will require upstream approaches to prevent the intoxicated person from drowning.

One-fifth (20%) of all fatal drownings in Australian rivers known to involve contributory levels of alcohol occurred in the early morning hours (i.e. 12:01 am to 6 am). It may be postulated that those more likely to consume alcohol in the afternoon and evening hours, be it at the river or not, are the ones who continue to drink alcohol into the early morning hours, increasing their risk of harm or injury, including drowning. While the link between risky drinking in everyday life and BACs ≥0.05% at the river was identified by this study, the assumption around time of day requires further testing to better illuminate the link between alcohol consumption, time of day and river drowning risk.

A limitation of this study was that survey and breathalysing data were not collected during the late evening and early morning hours, with the latest survey and breathalyser reading being recorded at 6:50 pm. However, numbers of river users decreased later in the day with the authors postulating that there would be very few river visitors after 8 pm at night. Alternative methods for collecting exposure and alcohol consumption-related data at rivers in both urban and regional areas during the late evening and early morning hours should be explored, and may include online surveys [28] and technological solutions such as remote camera observation [53], however the impact of time of year and season must be considered. Collecting BAC readings poses more of a challenge but remains worthy of further exploration.

### Boating

Aquatic location, activity being undertaken and exposure are all factors that may impact the likelihood and level of alcohol consumption. Unlike the other three research sites where recreating beside the water and swimming were the two main activities being undertaken, the Hawkesbury River site’s top two activities were boating (82%) and water skiing (55%). The Hawkesbury River site was also the only site where respondents were significantly less likely to record positive BACs (and therefore BACs ≥0.05%).

The potential link between participation in boating activity and decreased likelihood of alcohol consumption at rivers needs further examination. Length of stay at the river may be a factor. River users who self-reported participating in boating activities were significantly more likely to stay longer at the river (301+ minutes), however this study also found a link between staying longer at the river and likelihood of having a BAC ≥0.05% (121–300 min in the water X^2^ = 8.5; *p* = 0.007; 301+ minutes in the water X^2^ = 16.2; *p* = 0.002), which was not found among those participating in boating (X^2^ = 0.368; *p* = 0.544).

It may be that those participating in boating activities in the sample were less likely to drink due to needing to drive their motor vehicle to the boat ramp, the monetary value associated with their vessel and the impact of damaging it and also the perception of increased likelihood of being breath tested by police either on roads or the river, given the Hawkesbury River is located in an area defined as major cities. Further investigation with this cohort is vital, given that 24% of all fatal drownings due to boating and watercraft incidents were known to involve a person with a BAC ≥0.05% [5].

### Young males and risk taking

Drowning deaths of river users as a result of risk-taking behaviours (i.e. jumping into water from height) and alcohol are more likely to be young males [5]. This study did find a link between alcohol and self-reported risk taking behaviour, with those who agreed it was okay to drink alcohol as the skipper of a boat or while swimming in a river significantly more likely to record positive BACs and to drink at hazardous levels. Further research is required to better understand the link between alcohol and risk-taking behaviour, particularly among the young male cohort. Are young males aware they are taking a risk, do they indeed engage in risky behaviour because they enjoy taking risks and would such behaviour continue without the influence of alcohol? Further research is required to understand the psychological factors impacting such behavioural choices, which in turn will influence the development of strategies that are more likely to be effective in changing such behaviour [52].

Adolescence is described as an age of increased risk taking and impulsivity [54] and the published literature, often defines 16–21 year olds as the age group most likely to undertake risky behaviour [55] and to experience an escalation in alcohol use and misuse [56]. Due to ethical constraints, this study surveyed and breathalysed adults (18 years and over), and in reporting results, aggregated the 18–34 years age group to allow for comparison with previously published studies of alcohol-related river drowning and river exposure [5, 28]. The potential limitation of combining such disparate experiences within a heterogeneous age group must be considered and disaggregated in future studies to identify the ages of peak risk taking from an alcohol-related drowning prevention perspective. Further work is also required to examine underage drinking and the impact this has on drowning risk.

### Public holidays

It has long been postulated by drowning prevention researchers and practitioners that there may be increased risk of drowning on public holidays [57], due to opportunities for exposure to water as a result of more leisure time (adults not at work and children not at school) [58, 59], the celebratory nature of the occasion, and the consumption of alcohol [60]. This study found a link between the Australia Day public holiday and increased alcohol consumption at rivers, with a mean BAC among those who were consuming alcohol on Australia Day being 0.114%.

The site where data were collected on Australia Day had been designated an ‘alcohol free zone’ by the local council. However, as the data presented in this study shows, alcohol continued to be consumed, sometimes to excessive levels (e.g. the highest BAC recorded on Australia Day was 0.308%). The findings of this study have identified challenges around controlling safe alcohol consumption at public locations. Despite research showing public support for restrictions on alcohol consumption in public places [61], alcohol-free zones are unlikely to be effective without public awareness and enforcement of rules. Future questions to be answered include: Are alcohol-free zones likely to succeed in preventing all river users from drinking, or just those who do not drink at risky levels? Does it allow those who would drink to excess to ‘have the day off’ or does it move those who wish to drink to other, potentially less safe, locations to drink? What is the effect of such alcohol-free zones and are there other strategies to reduce alcohol consumption at rivers?

A limitation in being able to explore the link between public holidays, alcohol consumption and drowning risk, is that this data represents one public holiday at one aquatic location only. Further research is required to determine whether the phenomena is true of other public holidays, other rivers, and other types of aquatic location.

### Air temperature

The results of this study appear to indicate a link between hot weather and alcohol consumption. River users who were breathalysed on days with a maximum air temperature (36.8 °C–39.9 °C) were significantly more likely to record both positive BACs and BACs ≥0.05%. This finding may be used to guide the timing of prevention messages around alcohol risk and drowning in the lead-up to predicted high temperatures and the summer months.

The link between air temperature and drowning risk (not alcohol-related) has previously been explored. A study in Canada found a 69% increase in risk of outdoor drowning when temperatures exceeded 30 degrees Celsius [62]. While a study from Australia found air temperature did not impact beach visitation between genders, there was a slight impact on beach visitation by age group [63]. This impact of hot weather and alcohol-related drowning risk appears worthy of further testing, including the impact of temperature on both likelihood of consuming alcohol and amount of alcohol consumed at rivers, as well as other aquatic locations.

It must be noted that the maximum air temperatures reported in this study, do not take into account humidity. High humidity has the ability to dramatically increase how hot a day feels [64]. This is especially relevant to the Alligator Creek research site, which was located in northern Queensland. Capturing wet-bulb temperatures [65] to account for both air temperature and humidity should be incorporated into future studies examining alcohol consumption at rivers, although wet-bulb is not without its own limitations [66].

### Attitudes and behaviour

River users were significantly more likely to record a positive BAC and to drink at hazardous levels if they showed support for attitudinal questions around drinking alcohol while the skipper of a boat and drinking alcohol before swimming. Achieving attitudinal and behaviour change among this cohort is likely to prove challenging. Using established models around behaviour change such as the Transtheoretical model (TTM) [67], a model of behaviour change that focuses on the readiness of the individual to change their behaviour, allow for a starting point at which to develop appropriate strategies. The authors postulate that river users who consume alcohol at hazardous levels in their daily life are likely at the precontemplation stage and are unaware of the potential increased risk of drowning their drinking may create. Such assumptions require further validation.

The consumption of alcohol (often to excess) and participation in recreational activities in and around the water appear to be an intrinsic part of Australian culture [52, 68], meaning behaviours are deeply embedded and likely to take many years to change. A variety of strategies will be required to move people towards termination of consumption of alcohol at hazardous levels at rivers. Examining the psychological motivations underpinning such behaviours must form a vital component of any future research into river drowning and its prevention, to ensure appropriateness and efficacy of any intervention.

There were differences in attitudes towards acceptability of drinking and driving a motor vehicle and alcohol-related river usage among those surveyed. Of those surveyed, 7% agreed that it was okay to drink alcohol and drive a motor vehicle, 10% agreed it was okay to drink alcohol and operator a boat as skipper, 42% agreed it was okay to drink alcohol as a passenger on a boat and 21% agreed it was okay to drink alcohol before swimming. The authors posit such differences in attitude regarding alcohol use between road and river may be due to familiarity and understanding of the risks of drink-driving a motor vehicle due to exposure to advertising, as well as the visible police enforcement of legislation outlawing the behaviour through random breath testing, fines and prosecution [69]. While legislation already exists in seven of eight Australian states and territories (except the Northern Territory) regulating the operation of a powered vessel with a BAC ≥0.05%, enforcement is weak, in particular on rivers and outside metropolitan areas [5]. River drowning prevention practitioners should examine interventions that have been found to be successful in reducing injury due to alcohol in road traffic and explore if such strategies may be suitable for alcohol-related river drowning prevention.

### Strengths and limitations

Exposure around aquatic activity is challenging to capture. This study is the first of its kind and fills an important knowledge gap regarding exposure and consumption of alcohol at rivers. This study uses subjective measures (questionnaire) and objective measures (BAC reading) and cases of fatal unintentional alcohol-related river drowning to explore risk. While subjective measures have limitations, using the objective measure of a BAC reading confirmed a link between self-reported behaviour and a contributory level of alcohol. This study has identified river users at increased risk of alcohol-related river drowning and, therefore, targets for future interventions to change such risky behaviour.

Responses are self-reported and may be subject to recall bias [70], including questions on self-reported average daily alcohol consumption and alcohol consumption at ‘risky levels’ [71]. This is a limitation. Respondents may have also over-inflated their alcohol consumption when participating with their peers. As the research attracted media coverage (print, radio, television and online) the results may be subject to social desirability bias [72]. The survey was administered in English which may have impacted participation, particularly by those born outside Australia. The sample was a random convenience sample and therefore results represent the views of those attending the four river locations only. Caution should be used when extrapolating the results more broadly. Those in the study may have been subject to participation bias, with those more likely to drink, opting in; or those who didn’t drink, thinking the study was not applicable to them. The BAC reading represents a single point in time only. Further research is required to validate these findings more widely. A further limitation of this study was that data on refusal rate were not recorded. Although 3.6% of fatal drowning in rivers with contributory levels of alcohol occurred in children 17 years and younger [11], for ethical reasons, this study only included adults (18 years and older).

## Conclusion

Rivers are the leading location for fatal unintentional drowning in Australia and alcohol has been identified as a risk factor. A triangulation approach was taken using fatal river drowning statistics, surveying and breathalysing, to identify those at increased risk of alcohol-related drowning. Those at increased risk are: rivers users aged 18–34 years, residents of inner regional and low socio-economic areas, those who visit the river in the afternoon, with friends, and on days with higher maximum air temperatures, frequent river users (11+ times in the last 30 days) and those who spend longer in the water (301+ minutes). Prevention efforts should include targeting both males and females, and consideration of the role of warm weather, time of day, public holidays and those who consume alcohol to hazardous levels in daily life. This study addresses a gap in the published literature around river exposure and alcohol consumption.

## Additional files

Additional file 1: Understanding water safety at rivers. The full English language survey used in data collection for this study. (DOCX 13 kb)

Additional file 2: Table detailing characteristics of research sites, date of data collection, maximum air temperature and total daily rainfall Additional information about the four sites where data was collected for this study. (DOCX 82 kb)

## Abbreviations

ABS: Australian bureau of statistics
BAC: Blood alcohol concentration
CATI: Computer-assisted telephone instrument
HREC: Human research ethics committee
IBMSPSS: International business machines statistical package for the social sciences
IRSAD: Index of relative socio-economic advantage and disadvantage
TTM: Transtheoretical model
