# Supplementary material for: Breathalysing and surveying river users in Australia to understand alcohol consumption and attitudes toward drowning risk
Source: BMC Public Health. 2018 Dec 19;18:1393. doi: 10.1186/s12889-018-6256-1 (PMC6300037; doi:10.1186/s12889-018-6256-1)
Supplement: Supplementary file 1 — Understanding water safety at rivers. The full English language survey used in data collection for this study. (DOCX 13 kb) [file 12889_2018_6256_MOESM1_ESM.docx]

#
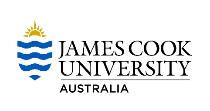

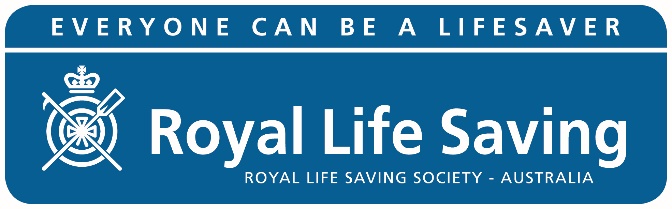


# Understanding water safety at rivers

#### 1) Do you give your informed consent to participate in this study?

[ ] Yes [ ] No - Please hand the survey form back to the researcher

## Demographics

#### 2) What is the postcode/zip code of your usual place of residence? _________________

#### 3) What is your gender? [ ] Male [ ] Female

### 4) What was your age at your last birthday? ___________________________

#### 5) What is your country of birth?

**[ ]** Australia

**[ ]**  Other - Write In: ________________________________________________

#### 6) Who are you with at the river/creek today? (tick all that apply)

[ ] Alone [ ] With friends [ ] With family

### 7) Including yourself, how many people are you here with at the river today? __________

## Frequency of river attendance

## 8) How many times in the last 30 days (month) have you visited THIS river (including today)? __________________

## 9) How many times in the last 30 days (month) have you visited ANY river (including today and this river)? __________________________________

## 10) How many times in the last 30 days (month) have you been to a beach? _______________

## 11) How far (in kilometres) did you travel to get to the river today? _____________________

## 12) Approximately what time did you arrive at the river today? Please use 24 hour time

## _________________________________________________

## 13) What time is it now? Please use 24-hour time _______________________________

## 14) How long have you been in the water today (approximately)? (in minutes) ____________

## 15) What time do you intend to leave? (Please use 24 hour time) ________________________

## Frequency of engaging in water activities

#### 16) Which of the following activities you have already done today or intend to do (you can choose both). Or if you don't intend to do it, or can't (e.g. can't swim, don't boat) please choose Not Applicable.

|  | **Already done today** | **Intend to do today** | **Not Applicable** |
| --- | --- | --- | --- |
| **Swim (including snorkelling/wading/floating/splashing)** | [ ] | [ ] | [ ] |
| **Swim alone** | [ ] | [ ] | [ ] |
| **Swim in water that was too deep to touch the bottom** | [ ] | [ ] | [ ] |
| **Swim at night when it is dark** | [ ] | [ ] | [ ] |
| **Swim within 2 hours of consuming alcohol** | [ ] | [ ] | [ ] |
| **Boating (including fishing from a boat)** | [ ] | [ ] | [ ] |
| **Boat within 2 hours of consuming alcohol (passenger/skipper)** | [ ] | [ ] | [ ] |
| **Walk/recreate/sit beside the water** | [ ] | [ ] | [ ] |
| **Jump into a river from a height (e.g. from a tree, bridge, rocks, or using a rope swing)** | [ ] | [ ] | [ ] |
| **Fishing from the river edge** | [ ] | [ ] | [ ] |
| **Paddle/canoe/kayak** | [ ] | [ ] | [ ] |
| **Dive into water of unknown depth** | [ ] | [ ] | [ ] |
| **Supervise children around water** | [ ] | [ ] | [ ] |
| **Water ski** | [ ] | [ ] | [ ] |
| **Camp (stay overnight)** | [ ] | [ ] | [ ] |

#### 17) Please rate how often you have done each of the following activities at any river in the past 12 months. If you can't do this activity (e.g. can't swim, no children to supervise) please choose Not Applicable.

|  | **1 - Never** | **3 - Sometimes** | **5 - Always** | **N/A - Don't do this** |
| --- | --- | --- | --- | --- |
| Swim (including snorkelling/wading/floating/splashing) | **[ ]** | **[ ]** | **[ ]** | **[ ]** |
| Swim alone | **[ ]** | **[ ]** | **[ ]** | **[ ]** |
| Swim in water that was too deep to touch the bottom | **[ ]** | **[ ]** | **[ ]** | **[ ]** |
| Swim at night when it is dark | **[ ]** | **[ ]** | **[ ]** | **[ ]** |
| Swim within 2 hours of consuming alcohol | **[ ]** | **[ ]** | **[ ]** | **[ ]** |
| Boating (including fishing from a boat) | **[ ]** | **[ ]** | **[ ]** | **[ ]** |
| Boat within 2 hours of consuming alcohol (passenger/skipper) | **[ ]** | **[ ]** | **[ ]** | **[ ]** |
| Walk/recreate/sit beside the water | **[ ]** | **[ ]** | **[ ]** | **[ ]** |
| Jump into a river from a height (e.g. from a tree, bridge, rocks, or using a rope swing) | **[ ]** | **[ ]** | **[ ]** | **[ ]** |
| Fishing from the river edge | **[ ]** | **[ ]** | **[ ]** | **[ ]** |
| Paddle/canoe/kayak | **[ ]** | **[ ]** | **[ ]** | **[ ]** |
| Dive into water of unknown depth | **[ ]** | **[ ]** | **[ ]** | **[ ]** |
| Supervise children around water | **[ ]** | **[ ]** | **[ ]** | **[ ]** |
| Water ski | **[ ]** | **[ ]** | **[ ]** | **[ ]** |
| Camp (stay overnight) | **[ ]** | **[ ]** | **[ ]** | **[ ]** |

#### 18) Have you ever driven through floodwaters? [ ] Yes [ ] No

#### 19) Have you ever swum in a flooded river? [ ] Yes [ ] No

#### 20) Which of the following best describes your current swimming ability?

[ ] I cannot float or swim

[ ] I can comfortably float and gently swim for up to 5 minutes

[ ] I can comfortably float and gently swim for up to 15 minutes

[ ] I can comfortably float and gently swim for up to 30 minutes

[ ] I can comfortably float and gently swim for over 1 hour

[ ] Unsure

#### 21) Rate your swimming ability compared with your peers

[ ] Poor – I can’t swim [ ] Below average compared with my peers

[ ] About the same as my peers [ ] Above average compared with my peers

[ ] Expert [ ] Unsure

## Drinking Patterns

#### 22) In the past year, how often did you have a drink containing alcohol?

[ ] Never [ ] Monthly or less [ ] 2-4 times a month [ ] 2-3 times a week

[ ] 4-5 times a week [ ] 6 or more times a week [ ] Don't know/Unsure

#### 23) How many drinks containing alcohol did you have on a typical day when you were drinking?

[ ] Zero - Don't Drink [ ] 1-2 drinks [ ] 3-4 drinks [ ] 5-6 drinks

[ ] 7-9 drinks [ ] 10 or more drinks [ ] Don't know

#### 24) Considering all types of alcohol beverages, how often did you have 4 or more (for females) or 6 or more (for males) drinks on one occasion in the past year?

[ ] Never [ ] Less than monthly [ ] Monthly

[ ] Weekly [ ] Daily or almost daily [ ] Don't know / Unsure

#### 25) In general, how often do you drink alcohol prior to visiting a river?

[ ] Never [ ] Sometimes [ ] Always [ ] Not Applicable

#### 26) In general, how often do you drink alcohol while at the river?

[ ] Never [ ] Sometimes [ ] Always [ ] Not Applicable

### 27) When you drink alcohol before visiting the river or while at the river, how many drinks do you USUALLY have? If you don’t drink, write 0 __________________________________

## Knowledge (Alcohol and Water Safety)

#### 28) When was the last time you undertook/updated first aid qualifications (including CPR)?

[ ] Never [ ] Completed for the first time in last 12 months

[ ] Updated in the last 12 months [ ] Completed/updated between 13 months and 2 years ago

[ ] Other - Write In: _________________________________________________

#### 29) Have you ever been rescued from a river? [ ] Yes [ ]No

#### 30) Have you ever been rescued at a beach? [ ]Yes [ ]No

## Attitudes (Alcohol and Water Safety)

#### 31) For each of the following statements please indicate if you strongly agree, agree, neither agree or disagree, disagree or strongly disagree. Ensure there is a response to EACH of the items.

|  | **Strongly agree** | **Agree** | **Neither agree or disagree** | **Disagree** | **Strongly disagree** | **Don't Know** |
| --- | --- | --- | --- | --- | --- | --- |
| Most drowning deaths are preventable | [ ] | [ ] | [ ] | [ ] | [ ] | [ ] |
| It is okay to drink and drive a motor vehicle | [ ] | [ ] | [ ] | [ ] | [ ] | [ ] |
| Holding your breath underwater is dangerous | [ ] | [ ] | [ ] | [ ] | [ ] | [ ] |
| It's okay to drink alcohol on a boat  (as a passenger) | [ ] | [ ] | [ ] | [ ] | [ ] | [ ] |
| It's okay to drink alcohol on a boat  (as the skipper) | [ ] | [ ] | [ ] | [ ] | [ ] | [ ] |
| It's okay to drink alcohol before swimming | [ ] | [ ] | [ ] | [ ] | [ ] | [ ] |

### Thank you for completing this survey, please hand the survey form back to the interviewer.

## Alcohol consumption

### 32) How long has it been since your last alcoholic drink? (e.g. 3 hours, 24 minutes etc) _________________________________________________

## BAC

### Interviewer to read: At the beginning of the interview you consented to a breath test as part of the survey. I would like to do this now… (…proceed with breath test…)

### 33) BAC reading (record to three decimal places e.g. 0.103) _________________________________________________

### 34) And the time is now (please record in 24 hour time) ___________________
