# Supplementary material for: Breathalysing and surveying river users in Australia to understand alcohol consumption and attitudes toward drowning risk
Source: BMC Public Health. 2018 Dec 19;18:1393. doi: 10.1186/s12889-018-6256-1 (PMC6300037; doi:10.1186/s12889-018-6256-1)
Supplement: Supplementary file 2 — Table detailing characteristics of research sites, date of data collection, maximum air temperature and total daily rainfall Additional information about the four sites where data was collected for this study. (DOCX 82 kb) [file 12889_2018_6256_MOESM2_ESM.docx]

Table detailing characteristics of research sites, date of data collection, maximum air temperature and total daily rainfall

| **Name of site** | **Remoteness classification of site** | **Description of site characteristics** | **Date of data collection** | **Maximum daily air temperature (degrees celcius) #** | **Total daily rainfall (mm)** |
| --- | --- | --- | --- | --- | --- |
| Alligator Creek, Townsville, Queensland | Outer Regional | Located within Bowling Green National Park, no gates or fee to enter. Carpark, BBQ facilities, covered tables for eating, public toilet block, boardwalk area, beach entry. One camping area with facilities and three camping areas without facilities. There is safety signage warning of previous death and injury at the site & disallowing glass. | Friday 12^th^ January | 31.7 | 0 |
|  |  |  | Saturday 13^th^ January | 31.5 | 0 |
|  |  |  | Sunday 14^th^ January | 33.5 | 0.2 |
| Murrumbidgee River, Wagga Wagga, New South Wales | Inner Regional | Carpark, BBQ facilities, covered tables for eating, public toilets and a child’s playground. A canoe club is located at the site. There is a grassed area with trees and a sandy beach entry to the river. Walkway past the beach entry and down further along the river. Safety signage was present warning of submerged objects, strong currents and deep water. | Friday 19^th^ January | 39.8 | 0 |
|  |  |  | Saturday 20^th^ January | 41.5 | 0 |
|  |  |  | Sunday 21^st^ January | 42.1 | 0 |
| Murray River, Albury, New South Wales | Inner Regional | Carpark, large public reserve, child’s playground, BBQ facilities, picnic tables, public toilets, public café (licensed – except for Australia Day). The river bank is grassed with concrete stairs and ramps to enter the river at certain points. There is safety signage warning about strong currents. The site was a designated ‘alcohol free zone’ on Australia Day. | Monday 22^nd^ January | 38.2 | 1.2 |
|  |  |  | Tuesday 23^rd^ January | 41.5 | 0 |
|  |  |  | Wednesday 24^th^ January | 33.5 | 1.8 |
|  |  |  | Thursday 25^th^ January | 35.8 | 0 |
|  |  |  | Friday 26^th^ January (Australia Day Public Holiday) | 37.3 | 0.6 |
|  |  |  | Saturday 27^th^ January | 34.2 | 3.4 |
|  |  |  | Sunday 28^th^ January | 36.8 | 0 |
| Hawkesbury River, Windsor, New South Wales | Major Cities | Carpark, large public reserve, public toilets, a boat ramp and a boardwalk area adjacent to the boat ramp at the rivers edge. The boat ramp featured safety signage regarding paddle craft, shallow water, wearing a lifejacket and alcohol. | Friday 2^nd^ February | 24.7 | 0.8 |
|  |  |  | Saturday 3^rd^ February | 26.8 | 3.2 |
|  |  |  | Sunday 4^th^ February | 28.6 | 4.0 |

# = Weather data sourced from climate data online from the Bureau of Meteorology (<http://www.bom.gov.au/climate/data/index.shtml?bookmark=136>) Access Date 20-03-2018].
